# Effects of Long-Term Administration of Lithium and Hydrochlorothiazide in Rats

**DOI:** 10.1155/MBD.1999.87

**Published:** 1999

**Authors:** Felicia Loghin, Adriana Olinic, Daniela-Saveta Popa, Carmen Socaciu, Sorin E. Leucuta

**Affiliations:** Department of Toxicology Faculty of Pharmacy University of Medicine and Pharmacy 13 Emil Isac Cluj-Napoca 3400 Romania

## Abstract

The biochemical and histological changes following 60 days administration of daily doses equivalent to 1/20 LD_50_ of lithium lactate and hydrochlorothiazide, as such and in association, were studied in male Wistar rats. No mortality or overt signs of toxicity were observed during the experiment and the serum activities of transaminases, alkaline phosphatase and cholinesterase were not significantly modified compared to controls. The histopathological examination of all the investigated organs: kidney, liver, brain and spleen, revealed significant lesions which were time-dependant and more pronounced in the association group. Although the changes were mostly inflammatory and conqestive, it was proved that the concomitant administration of lithium and hydrochlorothiazid is potentially dangerous, increasing lithium’s nephrotoxicity and the thiazide diuretic's hepatotoxicity.


### EFFECTS OF LONG-TERM ADMINISTRATION OF LITHIUM AND HYDROCHLOROTHIAZIDE IN RATS

Felicia Loghin*, Adriana Olinic, Daniela-Saveta Popa, Carmen Socaciu, and Sorin E. Leucuta

Department of Toxicology, Faculty of Pharmacy, University of Medicine and Pharmacy, 13 Emil Isac, 3400 Cluj-Napoca, Romania

### ABSTRACT

The biochemical and histological changes following 60 days administration of daily doses equivalent to 1/20 $LD_{50}$ of lithium lactate and hydrochlorothiazide, as such and in association, were studied in male Wistar rats. No mortality or overt signs of toxicity were observed during the experiment and the serum activities of transaminases, alkaline phosphatase and cholinesterase were not significantly modified compared to controls. The histopathological examination of all the investigated organs: kidney, liver, brain and spleen, revealed significant lesions which were time-dependant and more pronounced in the association group. Although the changes were mostly inflammatory and congestive, it was proved that the concomitant administration of lithium and hydrochlorothiazide is potentially dangerous, increasing lithium's nephrotoxicity and the thiazide diuretic's hepatotoxicity.

### INTRODUCTION

Lithium is a major drug in psychiatry, being used in the treatment and prophylaxis of bipolar affective disorder, but also in antidepressant-resistant patients (1,2,3). Lithium's pharmacokinetics is relatively simple, but the ion can pharmacokinetically interact with many usual drugs which may be prescribed for the treatment of an illness accompanying the psychiatric disorder: diuretics, nonsteroidal anti-inflammatory drugs, antibiotics, bronchodilators (4,5,6,7). The therapeutic index of the ion being low, such interactions may result either in inefficient levels or in toxic phenomena.

The thiazide diuretics have been recommended by some authors for the treatment of lithium-induced nephrogenic diabetes insipidus and in severe manic-depressive illness in which high doses of lithium do not produce therapeutic serum concentrations (8). The treatment can be efficient but also dangerous, due to a decrease in lithium's renal clearance.

Thiazide diuretics inhibit sodium reabsorption in the distal tubule, inducing sodium depletion. During long-term administration, the diuretic-induced sodium depletion results in a compensatory increased reabsorption of both sodium and lithium in the proximal tubule. Approximately 80% of lithium that has been glomerulary filtered is usually reabsorbed, but sodium depletion induces about 86% reabsorption (9). Therefore lithium concentrations can be significantly increased by diuretic therapy, with risk of intoxication.

In order to evaluate this risk, we have studied in male Wistar rats the biochemical and histological changes during long-term administration of lithium and hydrochlorothiazide as such and associated.

### MATERIALS AND METHODS

**Animals.** In the experiment were included 3 experimental groups and a negative control group, all four consisting of 30 male Wistar rats (body weight 122 ± 15 g). The animals were maintained in standard food and microclimate conditions during the whole period of observation.

**Dosing.** The substances were administered for 60 days, orally by intubation, lithium as lactate salt dissolved in distilled water and hydrochlorothiazide in aqueous suspension prepared with Tween 80.

The dosages were the following:

- experimental group I: a daily dose of lithium lactate equivalent to 1/20 $LD_{50}$ (105 mg/kg)

- experimental group II: a daily dose of hydrochlorothiazide equivalent to 1/20 $LD_{50}$ (309 mg/kg)

- experimental group III: a daily dose of 1/20 $LD_{50}$ of lithium lactate associated with 1/20 $LD_{50}$ of hydrochlorothiazide.

Negative controls received the same amount of Tween 80 in distilled water.

**Experimental Design**. Animals were checked daily for clinical signs and mortalities and weighed individually each week. On days 20, 40 and 60 of the experiment 10 rats from each group were sacrificed by decapitation, blood and the main organs: kidneys, liver, brain, spleen, heart and lungs being collected. The organs were weighted and their relative weights were determined. Serum was separated for lithium quantification by atomic absorption and for enzyme assay.

The serum activities of: transaminases (GOT, GTP), alkaline phosphatase (AP) and cholinesterase (ChE) were assayed. Analytical determination of transaminase activity was performed using a colorimetric method that measures for GOT activity the conversion of aspartic acid and α-ketoglutaric acid to glutamic acid and oxaloacetic acid, and for GPT activity the conversion of alanine and α-ketoglutaric acid to glutamic acid and pyruvic acid. The reaction product (oxaloacetic or pyruvic acid) reacts with dinitrophenylhydrazine to form a coloured hydrazone that can be determined by its absorbance in the visible range (505 nm)(10). Alkaline phosphatase was assayed using p-nitrophenylphosphate as substratum. The p-nitrophenol formed has an absorption maximum in alkaline medium at 405 nm (11). Serum cholinesterase was determined using as substratum butirylthiocholine. Thiocholine resulted in the presence of the enzyme reacts with 5,5'-dithiobis-2-nitrobenzoic acid to form a yellow product with an absorption maximum at 412nm (11).

Organ fragments of kidney, liver, brain and spleen were prepared according to the classical technique (10% formaldehyde fixation, paraffin embedded) and stained with haematoxylin-eosin for the microscopic examination, which was performed blindly.

**Statistical analysis.** The results are presented as mean values ± standard deviation and experimental groups were compared with negative controls by Anova analysis of variance and post-tests.

### RESULTS

No compound-related mortality or overt signs of toxicity were observed during the experiment.

### Serum Drug Analysis

Serum lithium concentrations during the experiment for lithium-treated groups were between 0.245 and 0.428 mmol/l after 20 days of administration, between 0.304 and 0.385 mmol/l after 40 days, and between 0.312 and 0.402 mmol/l after 60 days.

### Clinical chemistry

The evaluation of the enzymatic activities of transaminases and alkaline phosphatase revealed no significant variations from controls (Figures 1-3). Cholinesterase serum values are significantly decreased ($p < 0.05$) following 60 days of administration in the association group (Figure 4).

### Anatomic Pathology

***Organ weights.*** There were no compound-related organ weights findings. Slight variations between individual animals in both absolute and relative organ weights, which sometimes achieved statistical significance were observed, but were considered incidental and unrelated to the treatment.

***Necropsy findings.*** No macroscopic changes of the organs were observed.

***Histopathology.***

**Kidney**. After 2o days of lithium administration the changes consisted mostly in tubular dystrophy and a slight glomerular hypertrophy. After 40 days the tubular dystrophy, especially at proximal level, is more pronounced and a serous and fibrino-haemoragic glomerulitis can also be seen. The changes evolve in time, both at tubular and glomerular level and after 60 days the presence of a compact eosinophilic mass in the tubules can be observed, probably future hyaline bodies.

The administration of hydrochlorothiazide induced in 20 days a pronounced intra- and periglomerular blood stasis. The space between the visceral and parietal layer of Bowman's capsule is enlarged and tubular epithelial cells are modified. Cells showed irregular shape and surface alterations with extrusions reaching into the lumen. After 40 days the changes are more pronounced and early signs of serous glomerulitis are observed. At the end of the experiment, in the enlarged space of Bowman's capsule an eosinophilic mass is present. Tubular dystrophic changes are accompanied by incipient hyaline bodies formation.

The changes induced by the association of the two substances are earlier and more pronounced.

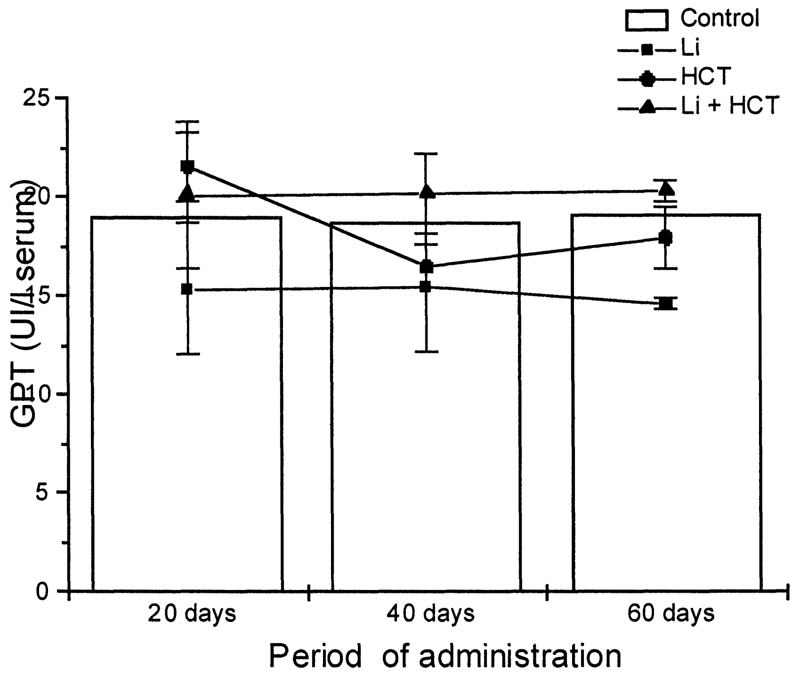

**Fig. 1. GPT activity variations during the experiment**

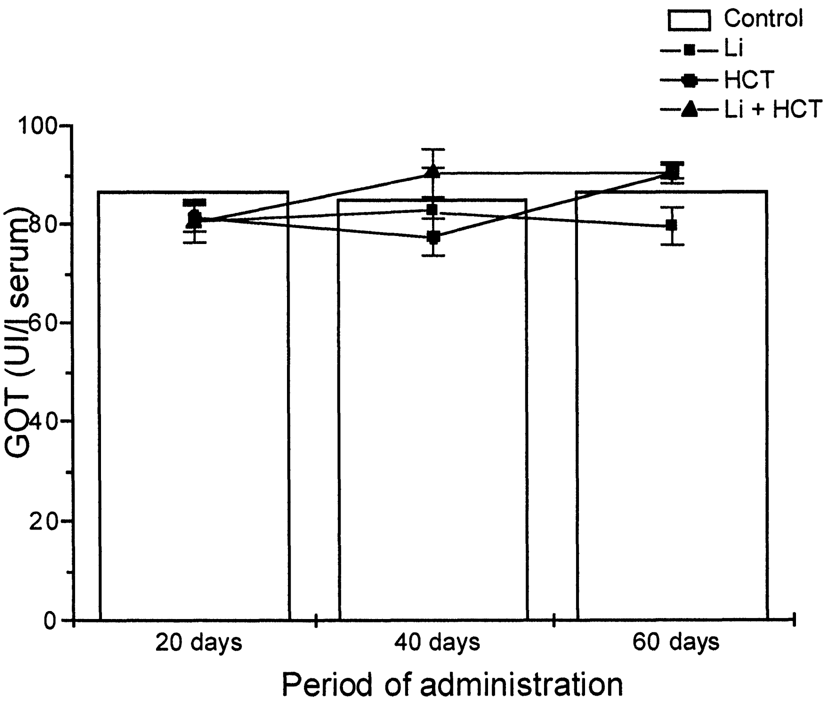

**Fig. 2. GOT activity variations during the experiment**

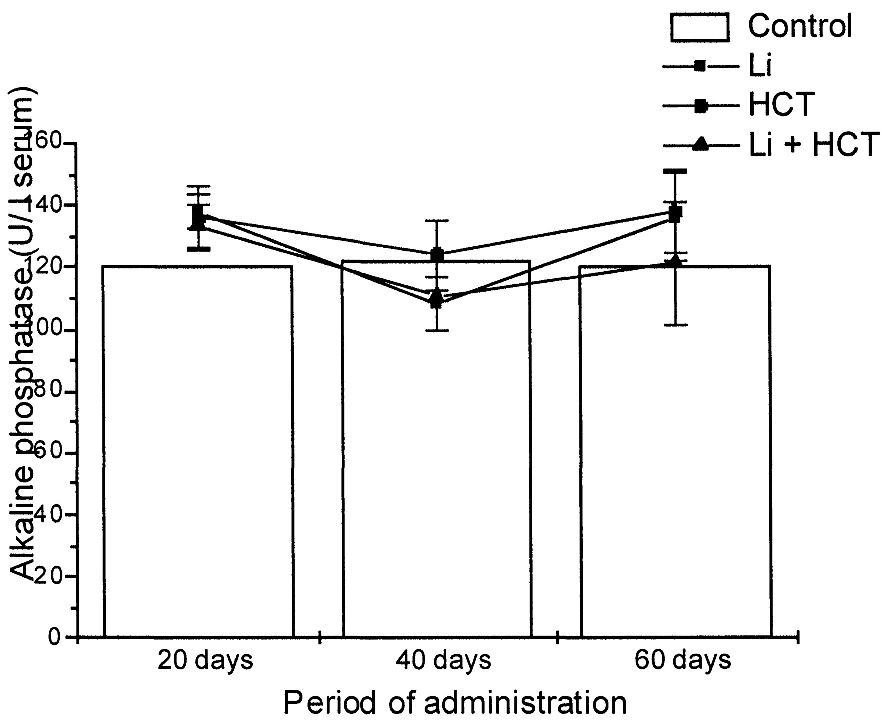

**Fig. 3. Alkaline phosphatase activity variations during the experiment**

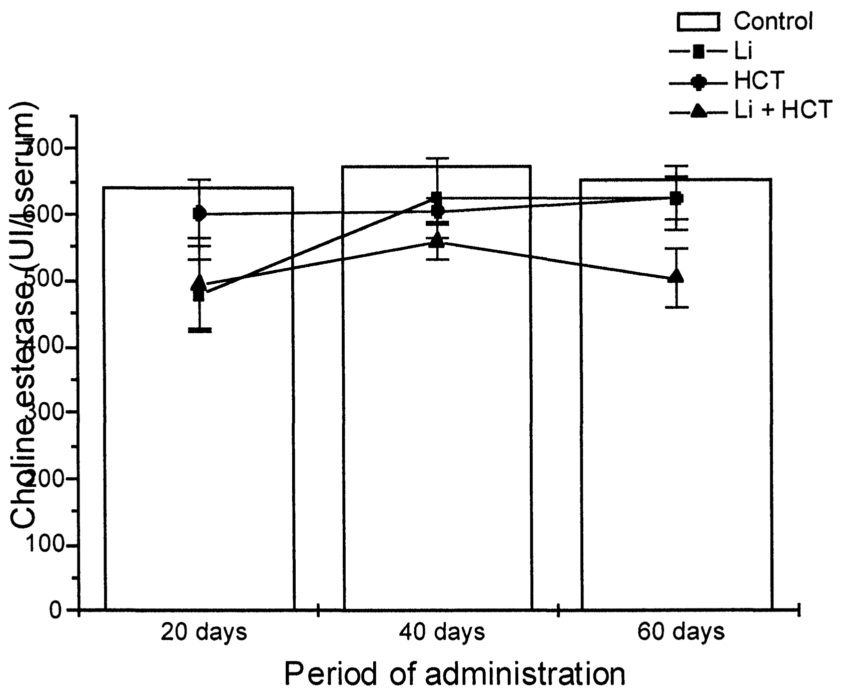

**Fig. 4. Choliesterase activity variations during the experiment**

Blood stasis is very pronounced from the beginning of the experiment. Both alterations of glomerular capillaries and the space of Bowman's capsule are of special interest. At day 40 of the experiment fibrino-haemoragic glomerulitis, glomerular hypertrophy and pronounced tubular dystrophy are observed. At the end of the experiment the interstitial, periglomerular and peritubular stasis are evident, the glomerular hypertrophy and the tubular dystrophy are very pronounced.

**Liver**. After 20 days of lithium administration, the sinusoid capillary dilatation and the presence of o diffuse lymphoplasmocytic infiltrate are seen. These become more evident in time, the structural disorganisation of the Remak cell columns and the presence of a nodule-like lymphoid infiltrate in the portal space being observed after 60 days.

Hydrochlorothiazide- induced hepatic changes are more pronounced and they become evident earlier. After 20 days, the structural disorganisation of Remak cell columns and even blood stasis in the portal space are observed. In time, an increase in binuclear hepatocyte number occurs and lymphoid nodules are present in the portal space. At the end of the experiment, the structural disorganisation and the enlargement of sinusoid capillaries are evident, the number of hepatocytes with cytoplasmic vacuolation is increased and a blood stasis is also present.

In the association group, the changes are similar but even earlier and much more pronounced. After 60 days the structural disorganisation associated with blood stasis, enlargement of sinusoid capillaries, increased number of binuclear hepatocytes and inflammatory nodules in the portal space are observed.

**Brain.** After 20 days of lithium administration, congestion and haemorrhage at meningeal level and a slight interstitial oedema. After 40 days, the interstitial oedema is more pronounced, a hyperplasia of the neurones and of the choroid plexus is observed. At the end of the experiment, the hyperplasia of the choroid plexus and the blood stasis are evident.

In hydrochlorothiazide group the changes are more pronounced, the hyperplasia of the choroid plexus is observed even after 20 days of administration and blood stasis and haemorrhage are already evident. After 40 days, the hyperplasia of the choroid plexus is more important and after 60 days a very pronounced interstitial oedema is present.

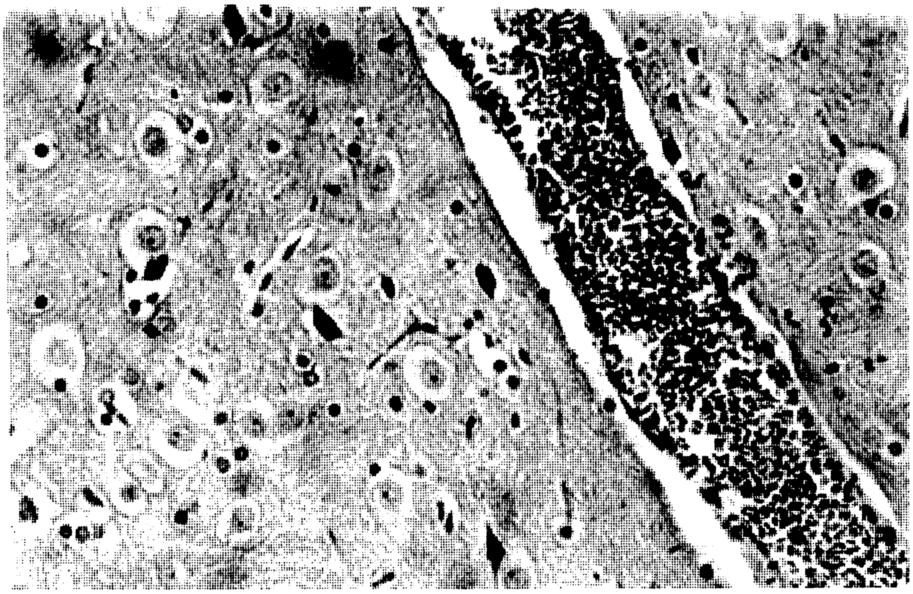

**Figure 5.** Brain, association group, 60 days, col.H.E.
Blood stasis and pronounced interstitial oedema

The association of lithium with hydrochlorothiazide potentiates the histological changes. The hyperplasia of the choroid plexus occurs earlier, as well as the interstitial oedema and after 60 days of administration the oedema is very pronounced and haemorrhage can also be observed (Figure 5).

**Spleen.** No significant changes were seen in lithium group at the beginning of the administration. But in time the white pulp becomes slightly hypertrophied and the red pulp has very few erythrocytes in the interstitium. After 60 days, the white pulp is hypertrophied and the red pulp is rich in lymphocytes and especially in macrophages.

In hydrochlorothiazide group, splenic changes can be observed even after 20 days of administration. The white pulp is hypertrophied and the periarteriolar lymphatic sheaths are thickened. The cords of Billroth in the red pulp are also hypertrophied and at the end of the experiment very few red blood cells and macrophages are present in the network.

The association induces progressive and more dramatic splenic changes. After 20 days the white pulp is slightly hypertrophied and the red pulp is rich in cells, with many red blood cells in the network. After 40 days the periarteriolar lymphatic sheaths begin to thicken. The changes are obvious after 60 days when in the white pulp thick periarteriolar lymphatic sheaths and in the red pulp thick cords of Billroth are observed. The red pulp is also very rich in red blood cells and macrophages.

### DISCUSSION

The results of the experiment pointed out that the long-term administration of lithium in rats results in toxic effects, although no mortality was observed.

Lithium serum concentration during the experiment did not attain levels between 0.6 and 1.2 mmol/ l which are considered toxic (12).

In these conditions, the activities of transaminases, alkaline phosphatase or cholinesterase, which are indicators of cytolysis, cholestasis or of the proteosynthetic function of the liver are not significantly modified. These results were confirmed by the histopathological examination which revealed only inflammatory, congestive or degenerative lesions. In the association group, where a hepatic structural disorganisation is observed, the activity of serum cholinesterase is decreased after 60 days.

Studies on patients following long-term administration did not confirm the hepatotoxic effect of lithium (13). Anyway, animal experiments revealed degenerative, dystrophic and congestive changes (14,15). As the concomitant administration of lithium and of drugs affecting the liver, like thiazide diuretics, results in an increased incidence of hepatic changes, a lithium hepatotoxic effect in these particular conditions should probably be taken into account.

The kidney is a major target organ for lithium's toxicity, and therefore highly investigated. Long-term animal studies revealed interstitial fibrosis, tubular atrophy and glomerular sclerosis (16,17). The renal changes observed during the present experiment were at the glomerular, interstitial and especially tubular level. The glomerular lesions can be both primary as well as secondary, due to tubular and interstitial changes. The severe tubular dystrophy is accompanied by sclerosis and it can be explained by the direct nephrotoxic effect of lithium. The changes are time-dependent and are more frequent at the association with hydrochlorothiazide, which in its turn can induce renal lesions. The thiazide diuretic, due to an increased tubular reabsorption, can increase lithium's concentration at kidney level (9). The elevated concentration may induce renal lesions, diminishing even more the elimination of the ion.

At the CNS level, congestion, haemorrhage and hyperplasia of the choroid plexus and of the neurones are the most striking phenomena. The changes have an increased incidence in the association group, not only due to an intrinsic toxic effect of the diuretic, but also to a 15% increase in lithium's brain concentrations as supported by previous research (18).

The splenic lesions are less important in lithium group, where they become evident only at the end of the experiment. The association with hydrochlorothiazide induces radical changes, with hypertrophy of both white and red pulp and an increase in the number of cells in the network: lymphocytes, macrophages and red blood cells.

### CONCLUSIONS

The long-term administration of lithium, as such and associated with the thiazide diuretic hydrochlorothiazide, induces toxic effects correlated to the period of administration. Even though no important behavioural or biochemical changes were observed, the renal, hepatic, cerebral and splenic lesions are significant. Although the kidney and in a greater extent the liver, have a certain regeneration capacity, and even in this case when the changes are mostly inflammatory, congestive and degenerative, the fact that they are more frequent in the association group raises the question of their irreversibility.

On the basis of these results, we can presume that the concomitant administration of lithium and hydrochlorothiazide increases lithium's nephrotoxic effect and the thiazide diuretic's hepatotoxicity.

**REFERENCES**

1) Hollister L.E., Csernansky J.G.. Clinical Pharmacology and Psychotherapeutic Drugs, Churchill-Livingstone Inc. New York (1990) : 132-133

2) Georgotas A., Cancro R.. Depression and Mania, Elsevier New York (1988) : 345-349

3) Austin M.P.V., Souza F.G., Goodwin G.M.. Lithium Augmentation in Antidepressant-Resistant Patients, Br.J.Psychiatry (1991) **159** : 510-514

4) Cazabat S.. Chez un patient sous lithium il faut se mefier constamment des interactions medicamenteuses, Le Quotidien du medecin (1993) **12** : 13-14

5) Ciraulo D.A., Shader R.I., Greenblatt D.J., Creelman W.. Drug Interactions in Psychiatry, Williams & Wilkins Baltimore (1987) : 127-151

6) Johnson F.N. Lithium Combination Treatment, Karger Basel (1987) : 246-260

7) Ragheb M.. The Clinical Significance of Lithium-Nonsteroidal Anti-inflammatory Drug Interactions, J.Clin.Psychopharmacol. (1990) **10**(5) : 350-354

8) Himmelhoch J.M., Poust R.I., Mallinger A.G., Hanin I., Neil J.F.. Adjustment of lithium dose during lithium-chlorothiazide therapy, Clin.Pharmacol.Ther. (1977) **22**(2) : 225-227

9) Young L.Y., Koda Kimble M.-A.. Applied Therapeutics. The Clinical Use of Drugs, Applied Therapeutics Inc. Washington (1988) : 1256-1263

10) Hayes W.A.. Principles and Methods of Toxicology, Raven Press New York (1982) : 413

11) Mitrica N.. Laboratorul Clinic. Biochimie, Ed.Medicala Bucuresti (1981): 283-300

12) Klemfuss H., Bauer T.T., Greene K.E., Kripke D.F.. Dietary calcium blocks lithium toxicity in hamsters without affecting circadian rhythms, Biol.Psychiatry (1992) **31**(3): 315-321

13) Viegut V., Jefferson J.W.. Lithium and the Liver, Liver (1990) **1**:9-13

14) Toader S., Kory M., Rotaru O., Pop R.D., Bohm B., Leucuta S.E.. Clinical Aspects and Histopathological Changes of Some Organs in the Intoxication Following Repeated Doses of Lithium Administered Intraperitoneally to Rats, Proceeding of 4 Spurenelement Symposium. Lithium. Jena (1983) : 300-305

15) Olinic A., Pantea A., Coca M., Onisor I., Anca Z., Kovats A.. Kidney, liver and thyroid changes in rats under the effect of long-term lithium excess, Clujul Medical (1994) **67**(3-4): 225-231

16) Marsussen N., Christensen S., Petersen J.S., Shalmi M.. Atubular glomeruli, renal function and hypertrophic response in rats with chronic lithium nephropathy, Virchows Archiv A . Pathol.Anat. (1991) **419**: 281-289

17) Fritz H. Lithium and the Developing Rat Kidney in Transplacental Target Organ Toxicity, Arzneim.Forsch./ Drug Res. (1988) **38**(1):50-54

18) Loghin F., Leucuta S.E.. Pharmacokinetic interactions of lithium with thiazide diuretics in rats, Farmacia (1994) **42** (5-6): 13-16

**Received: January 18, 1999 - Accepted: February 8, 1999 - Received in revised camera-ready format: March 8, 1999**

